# Adherence to iron-folic acid supplementation among pregnant women in Ethiopia: a systematic review and meta-analysis

**DOI:** 10.1186/s12884-020-2835-0

**Published:** 2020-03-04

**Authors:** Fikadu Waltengus Sendeku, Getnet Gedefaw Azeze, Selamawit Lake Fenta

**Affiliations:** 1 Department of Midwifery, College of Medicine and Health Sciences, Bahir Dar University, Bahir Dar, Ethiopia; 2 Department of Midwifery, College of Health Sciences, Woldia University, Woldia, Ethiopia

**Keywords:** Iron-folic acid adherence, Pregnant women, Systematic review, Meta-analysis, Ethiopia

## Abstract

**Background:**

Despite the supplementation of iron-folic acid is the recommended strategy during the antenatal period; iron deficiency anemia is the commonest hematologic complication during pregnancy. Therefore, this systematic review and meta-analysis aimed to assess the level of adherence to iron-folic acid supplementation and its associated factors among pregnant women in Ethiopia.

**Methods:**

Systematic review and meta-analysis guideline was followed for this study. Different online databases were used for the review: PubMed, HINARI, EMBASE, Google Scholar and African Journals Online. Different searching terms were applied based on the adapted PICO principles to achieve and access all the essential articles. The data were entered and analyzed using Microsoft Excel and Stata 11 software respectively.

**Results:**

Fifteen studies were included in this systematic review and meta-analysis with a total of 5808 pregnant women. The overall pooled prevalence of adherence to iron-folic acid supplementation among pregnant women in Ethiopia was 41.38% (95% CI: 33.09, 49.67). Having secondary and above educational status of the women (AOR:2.68,95%CI:1.25, 5.74), having an early registration of antenatal care follow-up (≤16 weeks) (AOR:2.54,95%CI:1.99, 3.24), having anemia complication during current pregnancy (AOR:3.01,95%CI:1.88, 4.81), having good knowledge of iron-folic acid supplementation (AOR: 2.96, 95%CI:1.76, 4.99), having four times or more antenatal care follow up (AOR:3.66, 95%CI:2.81, 4.77), getting health education about benefit of iron and folic acid (AOR:2.62,95%CI:1.46,4.72), and having good knowledge about anemia (AOR:2.99,95%CI:2.32, 3.85) were associated risk factors for adherence to iron-folic acid supplementation.

**Conclusion:**

The overall pooled prevalence adherence of IFAS among pregnant women was lower than the WHO recommendations. Educational status, early registration of ANC, anemia in the current pregnancy, good knowledge of IFAS, number of ANC visits, good knowledge of anemia and receiving health education about the benefit of IFAS were factors associated with the adherence of IFAS among pregnant women in Ethiopia. This finding is important to design strategic policies and to prevent anemia and congenital anomaly resulted from inadequate intake of iron and folic acid.

## Introduction

Iron-folic acid deficiency anemia is a public health concern worldwide, especially in low and middle-income countries. Iron with folic acid is an important micronutrient for physiological function, growth, and development as well as maintenance of life for the mother and her fetus during pregnancy and in later life. Similar to other nutrients, the demand, and constraint of iron and folic acid increases during pregnancy to meet the daily requirement for life development and growth of the fetus during pregnancy [1].

Non-adherence to iron-folic acid supplementation during pregnancy has a potential negative impact on the health of the mother and the fetus. Increased adherence to iron-folic acid supplementation during pregnancy was associated with reduced risk of anemia for the mother and hemorrhagic newborn disease and congenital anomalies for the fetus [2, 3].

Iron deficiency anemia is the commonest hematological disorder in pregnant women and children worldwide, particularly in low and middle-income countries [3–5].

Low intake of iron-folic acid during pregnancy has been associated with increased risk of adverse birth outcomes such as; neural tube defects, cardiac defect, and endocrine disorders. Iron-folic acid supplementation is currently the aforementioned and recommended strategies to prevent adverse birth outcomes and hematologic complications during pregnancy [6, 7].

Literature finding in South-East Asia, Latin America, and a few African countries showed that factors which lead to poor adherence to IFAS among pregnant women were; intolerance of gastrointestinal side effects that can occur with taking iron, inadequate supply of tablets, poor counseling of healthcare providers regarding the utilization of tablets and possible temporary side-effects to clients, inadequate utilization of prenatal health-care services, lack of awareness about the advantage of iron-folic acid and community believes, attitudes, and practices associated with women’s perception on up taking of iron-folic acid tablets [8].

Increased adherence to iron-folic acid for pregnant women enhance productivity and reduces iron deficiency anemia during pregnancy which minimizes the risk of hemorrhage, sepsis and maternal mortality and morbidity [9]. Low adherence hurts levels of energy, productivity, cognitive and physical development, and immune function [10]. Besides, inadequate intake of iron and folic acid during pregnancy has adverse neonatal outcomes such as; miscarriage, stillbirth, prematurity, low birth weight, congenital anomalies and perinatal morbidity and mortality [11].

In Ethiopia, iron-folic acid supplementation for 90 or more days is the main strategy for anemia control and prevention. However, adherence of iron-folic acid supplementation remains very low [12]. Therefore, this systematic review and meta-analysis aimed to estimate the pooled prevalence of adherence to iron-folic acid supplementation and its associated factors in Ethiopia.

## Methods

### Search strategy

This study was followed preferred finding items for systematic reviews and meta-analysis guideline. The databases used were; PubMed, HINARI, EMBASE, Google Scholar and African Journals Online. Adherence of iron with folic acid OR iron-folic acid compliance OR associated factors of adherence to IFAS OR during prenatal period OR antenatal period OR pregnant women OR pregnant mother AND Ethiopia and related were searched and used. Searching terms were based on adapted PICO principles to search through the above-listed databases to access all the essential articles.

### Reporting items

**Fig. 1:**
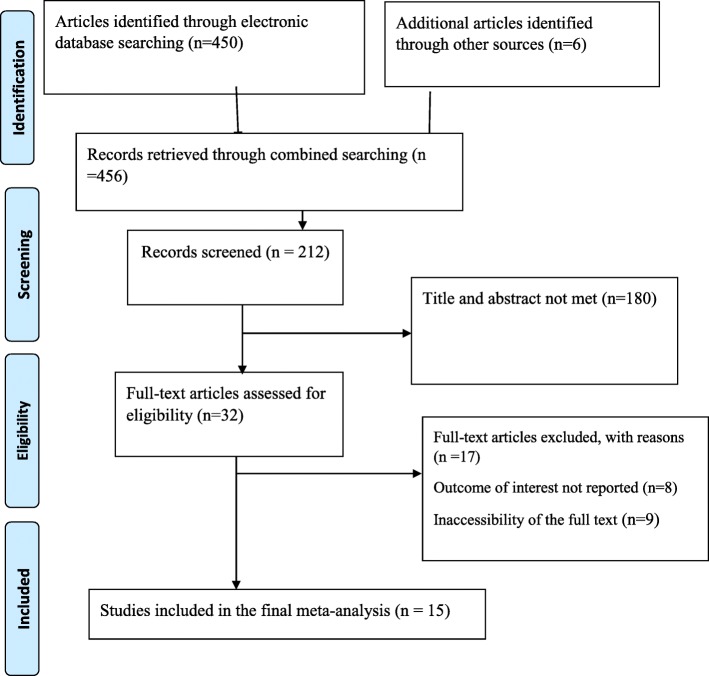
Flow chart of study selection for systematic review and meta-analysis of adherence to iron with folic acid supplementation among pregnant mother in Ethiopia

This study was designed according to the preferred reporting items for systematic review and meta-analysis Protocols (Fig. 1).

### Inclusion and exclusion criteria

This review was included articles which were cross-sectional study design and reported in English language. Articles reported prevalence and/or associated factors or determinant factors of adherence to iron with folic acid supplementation during prenatal period were included. Studies without full text and abstract, commentaries, letters, duplicated studies, anonymous reports, and editorials were excluded.

### Data extraction and quality assessment

After getting findings from all databases were exported to Microsoft Excel spreadsheet. Two authors (GG & FW) independently extracted the data and reviewed the screened articles. Any disagreement was handled by the third reviewer (SL). Finally, consensus was reached through discussion between authors. Newcastle-Ottawa Quality Assessment Scale (NOS) for cross-sectional study was used to assess the methodological quality of a study and to determine the extent to which a study has addressed the possibility of bias in its design, conduct, and analysis. All authors independently assessed the articles for inclusion in the review. All the included articles scored (NOS) 7 and more can be considered a “good” study and low risk.

### Outcome of measurement

This review has two main outcomes. Adherence of iron with folic acid was the primary outcome of the study whereas associated factors of adherence/compliance of iron-folic acid supplementation were the second outcome variable. The odds ratio was calculated for the common risk factors of the reported studies. The most common associated factors included in this systematic review and meta-analysis were educational status of the mother, early registration of ANC follow up before 16 weeks, frequent ANC visit 4 times or more, having knowledge of anemia, having a complication of anemia during current pregnancy, having knowledge of the advantage of iron with folic acid during pregnancy and getting health education about the advantage of iron with folic acid during pregnancy.

#### Adherent to IFAS

Pregnant women were reported how many times iron folic acid (IFA) supplementation tablets were taken per week by them from all prescribed tablets in the previous 1 month. In this case, pregnant women were considered as an adherent to iron folic acid supplementation if they were able to take at least 4 iron folic acid tablets per week in the previous 1 month preceding the survey [5].

### Publication bias and heterogeneity

Heterogeneity was checked using I^2^ and *p*-value. A value of 25, 50, and 75% was used to declare the heterogeneity test as low, moderate and marked heterogeneity respectively. Random effect model analysis was used with the evidence of heterogeneity. Funnel plot and Egger regression asymmetry test was used to check the existence of publication bias. Sub-group analysis and sensitivity analysis was employed to select the most influential risk factors with the evidence of publication bias.

### Data analysis

The data were entered and analyzed using Microsoft Excel and Stata 11 software respectively. Forest plots were used to report the estimated prevalence of each study with the 95% confidence interval (CI). The estimated pooled prevalence was computed with 95% CI. Subgroup analysis was computed using study setting and study area. The random-effects model was used to obtain the pooled odds ratio estimate if statistically significant heterogeneity (I^2^ ≥ 0) is evidenced; whereas, in case of no inconsistency in risk estimates (I^2^ = 0), a fixed-effect model was used. Finally, association of adherence of IFAS with educational status of mother, early registration for ANC follow up, history of current pregnancy anemia, knowledge of the benefit of IFAS during pregnancy, getting health education about the advantage of IFAS during pregnancy, having more than 4 times ANC visit and having good knowledge of anemia were computed with the evidence of odds ratio**.**

## Results

### Characteristics of the included studies

Four hundred fifty-six studies were retrieved at PubMed, HINARI, EMBASE, Google Scholar, African Journals Online, other gray literature’s and online repository accessed articles stating the level of adherence to iron-folic acid among pregnant women in Ethiopia. After duplication is expunged, 212 studies were remaining. Out of the remaining 212 articles, 180 articles were excluded after review of their titles and abstracts. Therefore, 32 full-text articles were accessed and assessed for inclusion criteria, which resulted in the further exclusion of 17 articles primarily due to reasons, 8 outcomes of interest not reported (*n* = 8) and inaccessibility of the full text (*n* = 9) (Fig. 1). As a result, 15 studies were met the inclusion criteria to undergo the final systematic review and meta-analysis.

### Study characteristics

Different maternal characteristics such as; educational status, antenatal care follow up, knowledge towards IFAS, and current pregnancy complication with anemia were included in this study. Fifteen cross-sectional studies with a total of 5808 pregnant women were included in this review. Amongst, fifteen studies eleven of them were conducted at facility-based and four of the studies were conducted at community-based study setting. Regarding the study area, eight of the studies were conducted at Amhara region followed by SNNPR (south nation nationalities and people representative) and Tigray region; each account three studies respectively (Table 1).

**Table 1:** Descriptive summary of fifteen included studies in the systematic review and meta-analysis

Authors	Year of study	Region	Study setting	Sample size	Prevalence of IFAS (%)
Abel et al. [13]	2015	Addis Ababa	Facility-based	557	60.1 (56.03,64.17)
Gashaw et al. [14]	2017	Amhara	Facility-based	418	28.7 (24.36, 33.04)
Gebremichael et al. [15]	2018	Tigray	Facility-based	222	10.5 (6.47, 14.53)
Habtamu et al. [16]	2018	Amhara	Facility-based	412	47.6 (42.78, 52.42)
Tsegaye et al. [17]	2018	Amhara	Facility-based	348	52.9 (47.66, 58.14)
Tarekegn et al. [18]	2016	Amhara	Facility-based	395	28.01 (23.58, 32.44)
Alemayehu et al. [19]	2017	Amhara	Facility-based	262	44 (37.99, 50.01)
Mekdemariam et al. [20]	2015	Tigray	Facility-based	320	64.7 (59.46, 69.94)
Tesfaye et al. [21]	2017	Amhara	Facility-based	418	55.3 (50.53, 60.07)
Zemenu et al. [22]	2015	SNNPR	Community-based	422	38.3 (33.66, 42.94)
Bekele et al. [23]	2013	Amhara	Community-based	634	20.4 (17.26, 23.54)
Abinet et al. [24]	2015	SNNPR	Community-based	303	39.2 (33.70, 44.82)
Asmamaw et al. [25]	2018	Amhara	Facility-based	422	43.1 (38.38, 47.82)
Negussie et al. [26]	2017	SNNPR	Community-based	317	51.4 (45.90, 58.90)
Afework et al. [27]	2014	Tigray	Facility-based	358	37.2 (32.09, 42.21)

### Iron-folic acid supplementation among pregnant women in Ethiopia

Pregnant women’s adherence to IFAS plays a key role in the prevention and treatment of iron-folic acid deficiency anemia. The World Health Organization recommends IFAS to all pregnant women in a standard dose of 30 mg–60 mg iron and 400 μg folic acid daily throughout pregnancy [9].

**Fig. 2:**
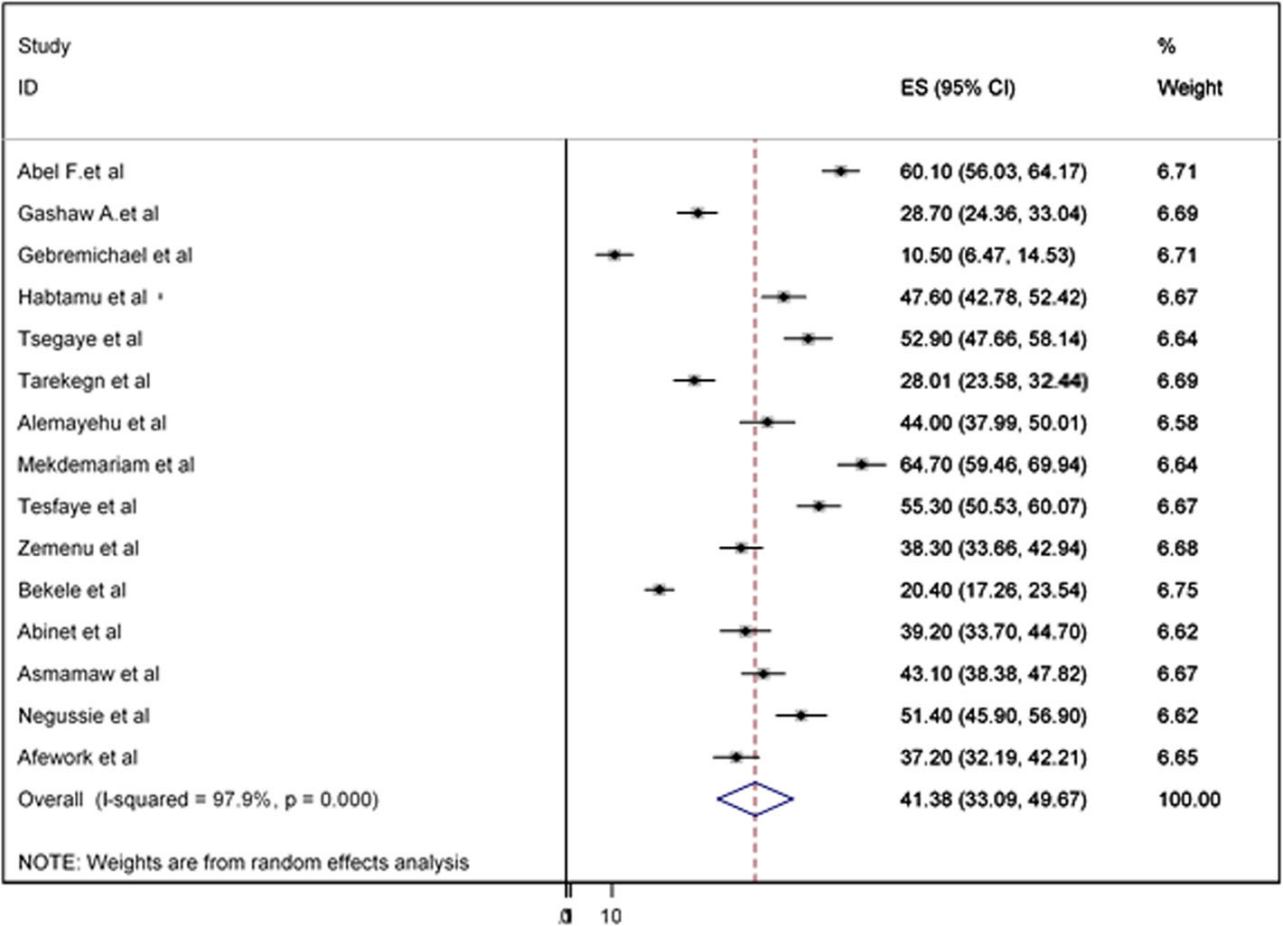
Forest plot of the pooled prevalence of iron and folic acid supplementation among pregnant women in Ethiopia

The overall pooled prevalence of adherence to iron-folic acid supplementation among pregnant women in Ethiopia was presented with a forest plot (Fig. 2). Therefore, the pooled estimated prevalence of adherence to iron-folic acid supplementation among pregnant women in Ethiopia was 41.38% (95% CI; 33.09–49.67; I^2^ = 97.9%, *P* < 0.001).

### Publication bias

**Fig. 3:**
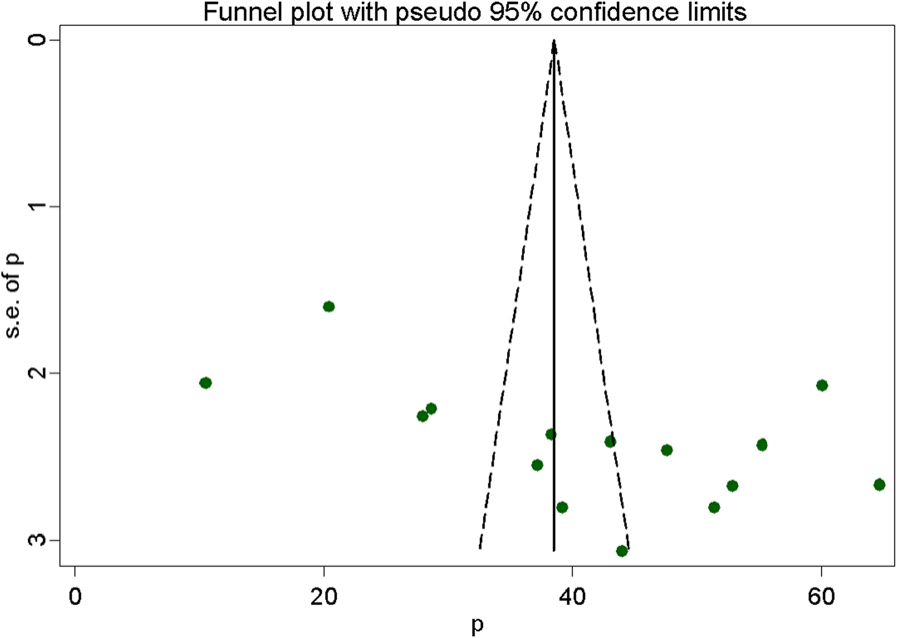
Funnel plot with 95% confidence limits of the pooled prevalence of adherence to iron and folic acid supplementation among pregnant women in Ethiopia

Funnel plot was assessed for asymmetry distribution of prevalence of adherence to iron-folic acid supplementation among pregnant women by visual inspection (Fig. 3). Egger’s regression test showed with a *p*-value of 0.02 indicated the existence of publication bias.

### Sensitivity analysis

**Fig. 4:**
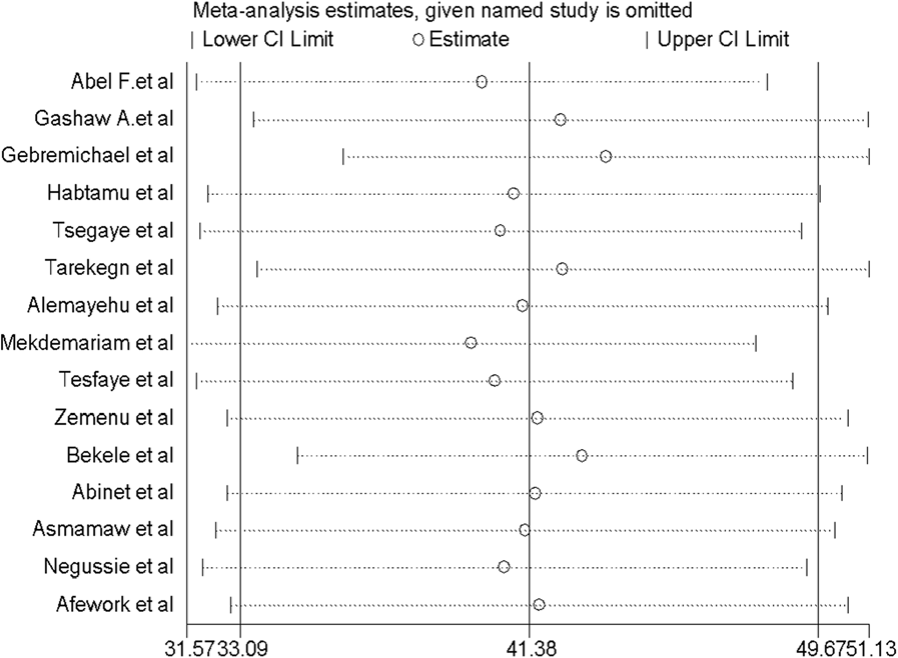
Sensitivity analysis of the pooled prevalence of adherence to iron and folic acid supplementation among pregnant women in Ethiopia

This systematic review and meta-analysis showed that the point estimate of its omitted analysis lies within the confidence interval of the combined analysis (Fig. 4).

### Subgroup analysis

Subgroup analysis was employed with the evidence of heterogeneity. Furthermore, the Cochrane I^2^ statistic =97.9%, *P* < 0.001) with evidence of marked heterogeneity, subgroup analysis was done using study setting and study area. Based on the subgroup analysis, the prevalence of adherence to iron and folic acid supplementation among pregnant women in Ethiopia was 42.89% (95%CI: 32.72–53.06, I^2^ = 97.4%, *P* ≤ 0.0001) were computed among studies conducted an institution (facility) based studies (Table 2).

**Table 2:** Sub group prevalence of adherence to iron and folic acid supplementation among pregnant women in Ethiopia

Variables	subgroup	No. of studies	Model	Prevalence (95%CI)	I^2^ (%)	*P* value
Study setting	Institution based	11	random	47.3 (36.25–58.36)	98	< 0.001
	community based	4	random	37.2 (23.21–51.22)	97.4	< 0.001
Study area	AA+SNNPR	4	random	47.3 (36.25–58.36)	95.2	< 0.001
	Amhara	8	random	39.9 (30.23–49.65)	97.3	< 0.001
	Tigray	3	random	37.4 (6.09–68.78)	99.2	< 0.001

### Risk factors for adherence to iron-folic acid supplementation among pregnant women in Ethiopia

In this systematic review and meta-analysis; educational status of the women, early registration antenatal care follow-up, having a complication of anemia during the current pregnancy, having a good knowledge of IFAS, having four times or more than antenatal care follow up, getting health education or counseling about the advantage of IFAS and having a good knowledge about anemia were factors associated with adherence to IFAS [13–27].

Having secondary and above maternal educational status (AOR: 2.68, 95%CI: 1.25, 5.74) was positively associated with the level of adherence to iron-folic acid supplementation. Moderate heterogeneity (I^2^ = 73.8%; *p*-value = 0.009) was detected among the included studies; for this reason, the random-effect meta-analysis model was computed. Furthermore, the existence of publication bias was detected using the Egger’s tests with a *p*-value of 0.034.

Early registration of antenatal care follows up (< 16 weeks) was the predictors of the level of adherence to iron-folic acid supplementation (AOR: 2.54, 95%CI: 1.99, 3.24). No heterogeneity (I^2^ = 0%; *p*-value = 0.596) was detected among the included studies; for this reason, the fixed-effect meta-analysis model was computed. Moreover, no possibility of publication bias was detected using the Egger’s tests with a *p*-value of 0.76.

Women with current pregnancy complications with anemia were positively associated and significant factors for adherent to iron and folic acid supplementation (AOR: 3.01, 95%CI: 1.88, 4.81). Moderate heterogeneity (I^2^ = 70.8%; *p*-value = 0.004) was detected among the included studies; for this reason, the random-effect meta-analysis model was computed. Moreover, no possibility of publication bias was detected using the Egger’s tests with a *p*-value of 0.253.

Women who have good knowledge of IFAS were 2.96 times more likely to adhere IFAS than those who have poor knowledge of IFAS (AOR: 2.96, 95%CI: 1.76, 4.99). Marked heterogeneity (I^2^ = 90.4%; *p*-value ≤0.001) was detected among the included studies; for this reason, the random effect meta-analysis model was computed. Moreover, the existence of the possibility of publication bias was detected using the Egger’s tests with a *p*-value of 0.001.

Woman who had four times or more than antenatal care follow up were 3.66 times more likely adhere to iron-folic acid supplementation than the counterparts (AOR: 3.66, 95%CI: 2.81, 4.77). No heterogeneity (I^2^ = 0%; *p*-value = 0.726) was detected among the included studies; for this reason, the fixed-effect meta-analysis model was computed. Moreover, there is no possibility of publication bias was detected using the Egger’s tests with a *p*-value of 0.787.

Women who have good knowledge of anemia were 2.99 times more likely to adhere with iron-folic acid supplementation than those women who have poor knowledge of anemia (AOR: 2.99, 95%CI: 2.32, 3.85). No heterogeneity (I^2^ = 0%; *p*-value = 0.525) was detected among the included studies; for this reason, the fixed-effect meta-analysis model was computed. Moreover, there is no possibility of publication bias was detected using the Egger’s tests with a p-value of 0.377.

Getting health education about iron and folic acid supplementation was associated with increased adherence to IFAS (AOR: 2.62, 95%CI: 1.46, 4.72). Marked heterogeneity (I^2^ = 84.7%; *p* < 0.001) was detected among the included studies; for this reason, the random effect meta-analysis model was computed. Moreover, possible publication bias was detected using the Egger’s tests with a *p*-value of 0.0001 (Table 3).

**Table 3:** Summary of associated risk factors for the adherence to iron and folic acid supplementation among pregnant women in Ethiopia

Variables	Model	Egger test (P-value)	Status of heterogeneity	AOR (95%CI)	I^2^ (%)	P value
Secondary & above maternal educational status	Random	0.034	moderate heterogeneity	2.68 (1.25, 5.74)	73.8	0.009
current pregnancy complication with anemia	Random	0.253	moderate heterogeneity	3.01 (1.88, 4.81)	70.8	0.004
Early registration of ANC (< 16 weeks)	Fixed	0.76	No heterogeneity	2.54 (1.99, 3.24)	0	0.596
Good knowledge on IFAS	Random	0.001	Marked heterogeneity	2.96 (1.76, 4.99)	90.4	≤0.001
Having four times or more than ANC	Fixed	0.787	No heterogeneity	3.66 (2.81, 4.77)	0	0.726
Good knowledge of anemia	Fixed	0.377	No heterogeneity	2.99 (2.32, 3.85)	0	0.525
Getting health education about IFAS	Random	0.0001	Marked heterogeneity	2.62 (1.46, 4.72)	84.7	≤0.001

## Discussion

In this systematic review and meta-analysis, the overall pooled prevalence of adherence to IFAS among pregnant women in Ethiopia was 41.38% (95%CI: 33.09, 49.67). The result of this study is in line with the study done in Pakistan, 38% of pregnant women adhere to IFAS [28]. The possible reason might be because of the similar socio-economic status of the women. Besides, both the studies were conducted with similar study settings and designs.

The finding of this systematic review and meta-analysis is lower than the study done in Southern Iran 51.47% [29], Senegal 51% [30], Iran 72% [31], Mozambique 79% [32], Sudan 65.4% [33] and Egypt 63.3% [34]. This discrepancy might be because of women living in urban areas, who might have a chance to access health services and awareness towards IFAS could increase the adherence of IFAS.

The finding of this study is higher than the studies conducted in Kenya 24.5% & 32.7% [35, 36], and Uganda 12% [37]. This difference might be because of the perception of the women is different across the countries due to different reasons such as; fear of side effects for IFAS, lack of awareness about the advantage of the IFAS during pregnancy, and having lower educational level of the women may reduce the ability to take iron and folic acid nutrients during pregnancy.

Secondary and above maternal educational status (AOR: 2.68, 95%CI: 1.25, 5.74) was positively associated with the adherence to iron and folic acid supplementation. This study finding is supported by the study done in Senegal [30], Sudan [33], India [38] and Iran [31]. This might be due to increasing the likelihood of the education status of women has a potential and crucial effect to increase the awareness and knowledge of pregnant women for iron-folic acid deficiency anemia and its consequences. Besides, education could increase women’s ability to easily access information’s disseminated through health professionals and media.

Pregnant women who started early antenatal care follow up (< 16 weeks) were 2.54 times the odds of adherence to iron-folic acid supplementation (AOR: 2.54, 95%CI: 1.99, 3.24). This finding is consistent with the study conducted in the Philippines [39]. The possible explanation might be due to pregnant women who booked early for ANC service and got repeated counseling could gain a better knowledge regarding the benefit of IFAS to her pregnancy outcome.

In this systematic review and meta-analysis women who had anemia during the current pregnancy (AOR: 3.01, 95%CI: 1.88, 4.81) were 3.01 times more adherent than their counterparts. This study finding is supported by the study conducted in Tanzania [40] and Iran [31]. This might be due to that women who had anemia needs therapeutic management of iron and have more chances to receive in-depth health education and counseling.

Women who had good knowledge of IFAS (AOR: 2.96, 95%CI: 1.76, 4.99) were 2.96 times more likely to adhere to IFAS than those who had poor knowledge of IFAS. This study finding is supported by the study done in Nigeria [41], Senegal [30] and Egypt [34]. This might be due to women having good knowledge that may improve their ability to adherence to IFAS in pregnancy.

Women having four times or more than antenatal care follow up (AOR: 3.66, 95%CI: 2.81, 4.77) were 3.66 times more likely to adhere to iron-folic acid supplementation than their counterparts. This study finding is supported by the study done in Kenya [35] and Uganda [37]. This is explaining that ANC is an important mode for the delivery of iron supplementation and increment of adherence. Hence, increasing the frequency of ANC visits are a good opportunity to increase the contact time between pregnant women and health providers. Thus, health providers can disseminate key information/messages, especially the benefits of IFAS supplementation.

Pregnant women who have good knowledge of anemia (AOR: 2.99, 95%CI: 2.32, 3.85) were 2.99 times more likely to adhere to iron-folic acid supplementation than women who have poor knowledge towards anemia. This study finding is in line with the study conducted in Nigeria [42] and Vientiane [43]. This is might be due to the reason that knowledge helps a woman to have a good perception of the advantage of taking iron-folic acid tablets.

Pregnant women who got health education about IFAS (AOR: 2.62, 95%CI: 1.46, 4.72) were 2.62 times more likely to be adherent to IFAS than those who didn’t get health education. This finding is consistent with the study done in Senegal [30] and Uganda [37]. The possible justification might be due to getting health education may increase the level of knowledge, attitude and practice towards IFAS adherence to pregnant women.

### Limitation

This meta-analysis was well-thought-out only articles conducted in the English language, which may have restricted some papers from being included. In addition, all of the included studies were cross-sectional; as a result, the outcome variables might be affected by other confounding variables in nature and temporal cause and effect relationship may not be well addressed via cross-sectional studies.

## Conclusion

The overall pooled prevalence adherence of IFAS among pregnant women was lower than the WHO recommendations. Educational status, early registration of ANC, anemia in the current pregnancy, good knowledge of IFAS, number of ANC visits, good knowledge of anemia and receiving health education about the benefit of IFAS were factors associated with the adherence of IFAS among pregnant women in Ethiopia. This finding is important to design strategic policies and to prevent anemia and congenital anomaly resulted from inadequate intake of iron and folic acid. Therefore, iron and folic acid deficiency anemia can be prevented by delivering and implementing strategies to improve the adherence of iron and folic acid supplementation.

## Data Availability

All related data has been presented within the manuscript. The dataset supporting the conclusions of this article is available from the authors on request.

## Abbreviations

AA: Addis Ababa
ANC: Antenatal Care
CI: Confidence Interval
IFAS: Iron–Folic Acid Supplementation
OR: Odd Ratio
SNNPR: Southern nation’s nationality, people’s regional state
WHO: World Health Organization
